# *Azolla pinnata* for livestock: a sustainable protein with economic promise

**DOI:** 10.1007/s11250-025-04674-3

**Published:** 2025-10-27

**Authors:** Omnia Y. Abd-Elfadiel Hagag, Muhammed Ahmed-Hilmy El-Rayes, Ahmed S. El-Hawy, Zhour I. Nabil, Nahla Soliman El-Shenawy

**Affiliations:** 1 Department of Zoology, Faculty of Science, Suez Canal University, Ismailia, 4153 Egypt; 2 Animal and Poultry Physiology Department, Desert Research Center, Cairo, Egypt; 3 Department of Animal Physiology, Animal and Poultry Production Division, Desert Research Center (DRC), Cairo, Egypt

**Keywords:** Azolla, Animal feed, Protein source, Sustainable agriculture

## Abstract

The increasing demand for high-quality animal feed, particularly protein sources, presents a significant challenge in Egypt and other developing nations. Soybeans, the primary protein source in livestock production, have become less sustainable due to rising costs and global supply constraints. Consequently, the search for alternative protein sources has intensified, particularly for poultry and livestock feed. Among the promising alternatives, aquatic plants such as Azolla have gained considerable attention due to their high nutritional value, ease of cultivation, and potential for environmental sustainability. Azolla, a fast-growing, protein-rich aquatic fern, is a viable alternative protein source due to its high digestibility and balanced amino acid composition. *Azolla pinnata*, in particular, stands out for its affordability, adaptability, and diverse applications. It thrives in stagnant water bodies and has been traditionally used as green manure and biofertilizer. Recent studies have demonstrated its potential as a sustainable feed supplement for various livestock, including poultry, cattle, sheep, goats, and pigs. With a 25–30% protein content and a superior amino acid profile, *A. pinnata* can enhance animal growth and productivity while reducing reliance on conventional protein sources, such as soybean meal (Hassen et al. (Ethiop J Appl Sci Technol 10(2):22–28, 2019)). Azolla’s benefits extend beyond nutrition. It contributes to environmental sustainability by mitigating ammonia volatilization, improving soil fertility, and serving as a bioremediation agent in wastewater treatment. Its rapid biomass accumulation and nitrogen-fixing capability make it an economically viable feed option. Incorporating Azolla into livestock diets has been shown to enhance feed conversion efficiency, reduce feed costs, and improve animal health and productivity. Studies indicate that Azolla supplementation can increase milk yield in dairy cattle, improve weight gain in goats and sheep, and enhance poultry performance without adverse effects on animal health. Given its potential to address the global protein shortage and reduce dependence on costly imports, Azolla presents a promising solution for sustainable livestock production. Further research and strategic integration of Azolla into feeding systems could optimize its utilization and unlock new opportunities for enhancing food security and environmental sustainability in developing regions.

**Graphical Abstract:**

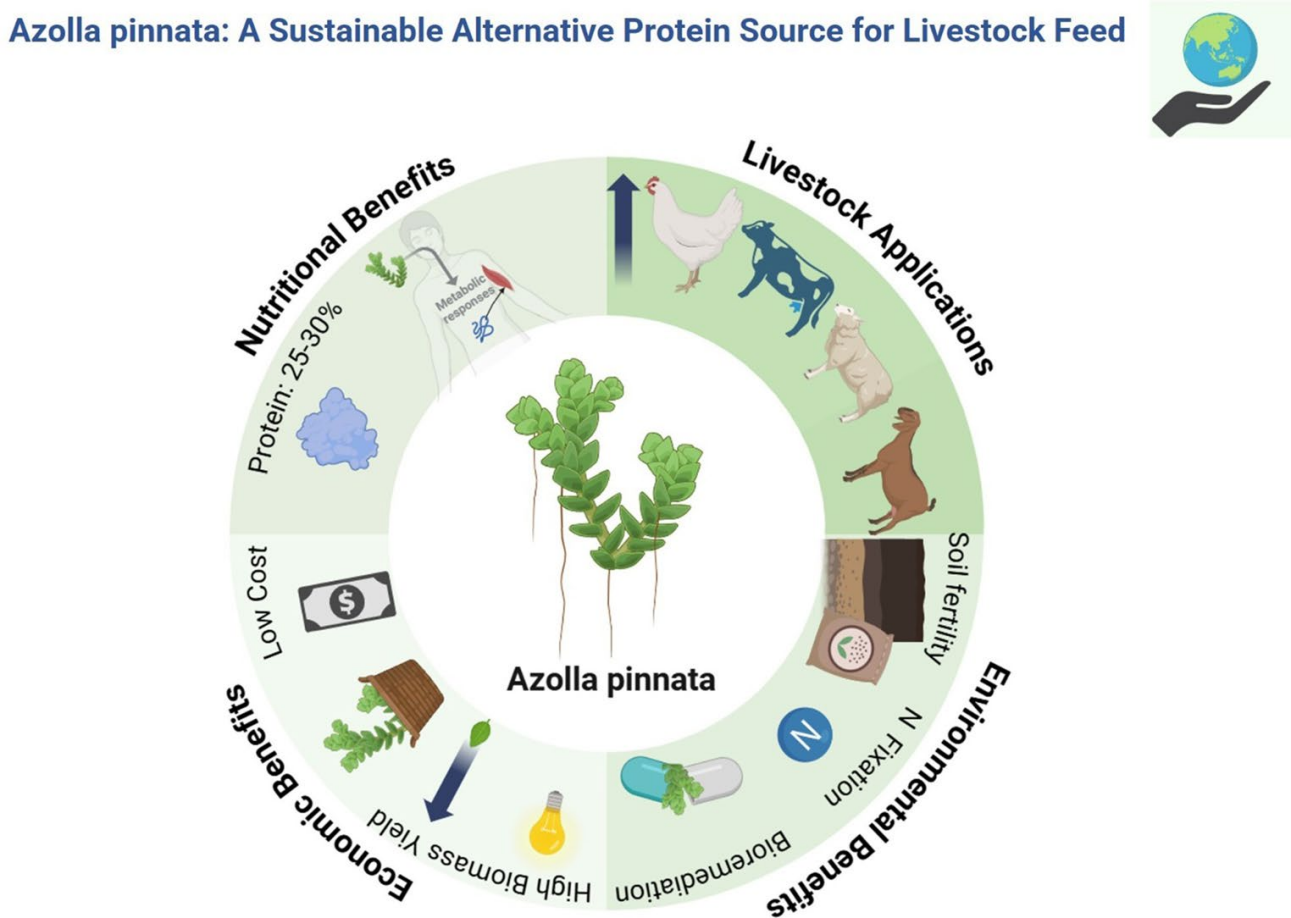

## Introduction

The shortage of animal feed, particularly protein sources, remains a significant challenge in Egypt and other developing nations (Badawy 2014); (Hassen et al. 2019). Soybeans are the most commonly used protein source in livestock production worldwide. However, as global food supplies dwindle and costs continue to rise, identifying alternative feed resources has become increasingly essential, particularly for poultry production (Mottet and Tempio 2017). Over the past decade, the reliance on a limited number of crops for food, feed, and energy production has intensified. Modern agricultural systems face sustainability challenges due to factors such as climate change, shrinking arable land, population growth, freshwater scarcity, and the high costs of electricity and chemical fertilizers (Tran et al. 2020). To address these issues and ensure a more affordable protein supply, there is a growing need to explore diverse protein sources.

Aquatic plants, including free-floating species such as duckweed and Azolla, have garnered interest due to their unique properties. They offer benefits similar to microalgae while being easier and more cost-effective to harvest (Pereira 2017). Unlike tree leaves, aquatic plants typically do not accumulate secondary plant compounds, making them a more viable protein source for monogastric animals (Abd El-Ghany 2020; Herath et al. 2023). The use of aquatic plants for various applications is drawing increased attention. Azolla, a genus of aquatic ferns commonly found in lakes, ponds, ditches, and other water bodies in warm climates, is particularly valued for its ease of cultivation, high productivity, and rich nutritional profile (Sadek et al. 2010). Traditionally, it has been utilized as green manure and biofertilizer in rice fields. More recently, both fresh and dried Azolla have been incorporated into livestock feed for ruminants and non-ruminants alike (Verma et al. 2022). Due to its high nutritional value, ease of cultivation, and minimal adverse effects on animal health and performance, it is considered a promising alternative protein source (Naser and Al-Musawi 2020).

Among the various Azolla species, *Azolla pinnata* is the most notable due to its affordability and ease of cultivation. It thrives naturally in stagnant water bodies such as canals, ponds, drains, rivers, and marshy areas, particularly in tropical and subtropical regions (El-Fadel et al. 2020). Its ability to absorb and concentrate minerals, nutrients, and other contaminants makes it highly effective for wastewater treatment and environmental remediation.

Often referred to as a “green goldmine,” *A. pinnata* is valued not only for its high nutritional content but also for its diverse applications. It has been utilized for weed control, ammonia volatilization reduction, and as a bioresource for biogas and hydrogen fuel production. Additionally, it has emerging applications in human nutrition, pharmaceuticals, and as a feed supplement for livestock due to its protein-rich composition (Kathirvelan et al. 2015). Despite its low oil content, *A. pinnata* boasts a substantial nutritional profile, containing 25–30% protein, 10–15% minerals, 7–10% carbohydrates, biopolymers, bioactive compounds, and amino acids.

Its amino acid composition further enhances its value as a feed ingredient. Notably, its essential amino acid content, particularly lysine, is 0.42% higher than that of conventional feed sources such as broken rice concentrate, bran, and corn, making it a superior protein alternative in animal diets (Korsa et al. 2024). Given its nutrient density, rapid growth, and wide-ranging applications, *A. pinnata* holds excellent promise as a sustainable resource for both agricultural and industrial purposes (Kouchakinejad et al. 2024).

This review highlights *A. pinnata* as a nutritionally rich, economically feasible, and environmentally sustainable alternative to conventional livestock protein sources, such as soybean meal. It stands out by synthesizing recent evidence on *A. pinnata*’s broad applicability across various livestock species, its superior amino acid profile, and its added value in reducing feed costs and improving environmental outcomes. Unlike prior fragmented research, this study provides a comprehensive analysis of *A. pinnata*’s dual role in enhancing livestock productivity and contributing to sustainable agriculture, particularly in resource-constrained regions like Egypt.

## Azolla is a sustainable protein

The nutritional needs for optimal growth, optimal return, minimal waste, and good health should all be met by high-quality feeds (Catherine and Amalaranis, 2016). The two most widely used protein sources by animal producers worldwide are soybeans and maize (Dei 2011). Recently, post-extraction soybean meal is utilized as a basic protein feed for poultry and is widely employed to feed a variety of farm animal species (Świątkiewicz et al. 2021). Soybean crops have a fresh weight content of about 18% fats and 40% proteins (Taelman et al. 2015). The expansion in the demand for plant protein by the expanding livestock sector has resulted in a 71% shortage in high-protein agricultural commodities, 87% of which is met by imported soybeans or local soybeans (Hamidan et al. 2024). Whereas the price of basic protein feedstock has increased significantly in recent years in comparison to the demand worldwide (Abdalbakee and Mohammed 2019), low economic productivity in some countries due to increased import costs is still ongoing if the future still depends on the same sources of protein, i.e., soybeans and maize (Nisar et al. 2024). However, farmers and feed producers were forced to cut back on or substitute other ingredients for soybean meal in their feed formulation because of the unaffordable prices. An attempt was made to find a different, viable, and reasonably priced source of protein and fiber for farmers (Hamidan et al. 2024).

Adopting sustainable, financially and socially viable strategies is crucial in light of the current situation (Kour et al. 2024). The United Nations Development Programmer’s (UNDP) and Sustainable Development Goals (SDGs) have been established for numerous nations, and are incompatible with a reliance on imported animal feed (Bahri et al. 2019). The world’s ecology is now in danger due to the intense agricultural methods used to satisfy the demands of an expanding population. When agrochemicals are used improperly to increase productivity, soil fertility, biodiversity, and the climate are all negatively impacted (Baweja et al. 2020). In the field of study, the SDGs are essential for enhancing natural resources and ensuring the environment’s long-term sustainability (Rehman et al. 2022, 2024).

Azolla can double its biomass in 5.6 days and is a very productive plant (Kösesakal and Yıldız 2019). Moreover, shows affordability, flexibility, and cost-effectiveness in both regulated farming situations and natural (non-cultivated) settings (Saikia et al. 2014). It produces an unusual symbiotic association with filamentous cyanobacteria, such as Anabaena and Nostoc, which subsequently fix nitrogen (Brouwer et al. 2017) (Table 1). Utilizing the organic matter generated by nitrogen-fixing plants to create organic fertilizers is a smart move, and Azolla is one possible way to achieve this (Maham et al. 2018) because they are easy to maintain, harvest, produce a lot of biomass, and have a high growth rate. Because of the phytoremediation potential of these plants, they can be utilized to enhance the quality of water (Kösesakal and Yıldız 2019). It, a well-known biotechnology product with potential for worldwide use, is an aquatic fern that is concerned with environmental sustainability (Nasir et al. 2022a). It has practically all of the essential amino acids and minerals, including iron, calcium, magnesium, potassium, phosphorus, manganese, and protein (Patil and Patil 2020). The quick colonization of Azolla on water surfaces makes it a common invasive weed. It is a free-floating aquatic fern that can fix nitrogen (Table 1), double quickly, and bioremediate (Arora et al. 2022). The low amount of aflatoxin (0.01 µg/kg) found in *Azolla sp*. is confirmed by a study by Shambhvi et al. (2021). Because of this, it can be fed to animals without hurting their health.

**Table 1:** Some Azolla species, their chemical composition, and importance

Azolla Species	Chemical Composition	Importance	Reference
A. ***pinnata***	High in nitrogen (~ 3–5%), protein (~ 25–30%), phosphorus, potassium, calcium, and amino acids. - Dry Matter: 86.72% - Crude Protein: 26.46% - Crude Fiber: 13.67% - Ether Extract: 3.92% - Nitrogen-Free Extract: 41.97% - Total Ash: 24.49% - Organic Matter: 76.19% - Rich in minerals like calcium, phosphorus,	Used as a biofertilizer, animal feed, and wastewater treatment agent	Gangadhar et al. (2015); Tadavi et al. (2017); Brouwer et al. (2018); Gupta et al. (2018); Verma et al. (2022)
A. ***filiculoides***	Rich in nitrogen (~ 4–5%), proteins, lipids, carotenoids, Nitrogen Content: 0.74% (wet weight) - Contains essential amino acids, vitamins, and minerals	Effective in nitrogen fixation, carbon sequestration, and bioremediation	Bhaskaran and Kannapan (2015); Brouwer et al. (2017); Arora et al. (2022); Rahman et al. (2023)
A. ***microphylla***	Contains ~ 4% nitrogen, 25–30% crude protein, vitamins, and minerals	Used in paddy fields as a natural fertilizer and in livestock feed	Querubin et al. (1986); Fiogbe et al. (2004); Korsa et al. (2024)
***A. caroliniana***	Rich in nitrogen, iron, phosphorus, flavonoids, and polyphenols	Used for soil enrichment, water purification, Potential animal feed supplement, and Phytoremediation capabilities	Kalita and Borah (2013); Nasir et al. (2022a, b); Rahman et al. (2023)
***A. mexicana***	High protein (~ 20–30%), nitrogen, and essential nutrients	Used as feed for fish and poultry and in phytoremediation	Rashad (2021)
***A. nilotica***	Moderate nitrogen content (~ 3–4%), essential minerals	Primarily used for composting and organic farming	Ebrahim et al. (2007)

The chemical composition of Azolla species can vary based on environmental conditions, cultivation methods, and processing techniques

## Nutritional value and utilization of Azolla in livestock feed

Proteins are the primary building block of muscle tissue and stimulate a variety of biological processes (Damodaran 2008). Leafy plant material is high in protein because it is also necessary for photosynthesis. The bulk protein in our feed and food comes from stored proteins found in seeds, tubers, and other plant storage organs that support plant reproduction (Kirkby et al. 2023). Animal growth has been demonstrated to be improved by Azolla, depending on the amount of the animal’s feed that it contains. *A. pinnata* is the most widely utilized species of Azolla for livestock feed (Nasir et al. 2022b). Azolla’s value as feed or fodder for fish and cattle is mostly determined by its amino acid content, which is followed by other substances, including crude protein, fiber, and digestible carbohydrates (Roy et al. 2016). For both humans and animals, amino acids are typically categorized as either nutritionally necessary (indispensable) or nonessential (dispensable) depending on growth or nitrogen balance, specifically the net synthesis of protein throughout the body (Wu 2009). Up to 18 amino acids, such as glutamic acid (12.6% protein), aspartic acid (9.3%), leucine (8.4%), alanine (6.4%), arginine (5.9%), glycine (5.6%), and valine (5.5%), are found in Azolla (Roy et al. 2016). Additionally, it is a protein-rich, amazing plant found in nature. It also contains almost all of the essential minerals, vitamins, and amino acids, including B12 and carotene, which is a precursor to vitamin A, while having relatively low levels of fat and carbohydrates. There are fewer risks and less work involved in using aquatic ferns (Azolla) (Korsa et al. 2024).

Azolla is a free-floating fern that grows all over the world. The plant is composed of a slender, branched, floating rhizome with tiny leaf-like fronds and roots that reach deep into the water. In the context of a changing climate, temperature-tolerant plants are acknowledged as a crucial research field to be examined since temperature is one of the major climatic elements influencing plant growth (Korsa et al. 2024). However, conditions like water temperature, light intensity, and nutrition availability can affect the precise yield (Golzary et al. 2021). The wide range of chemical compositions found in these experiments indicates that the strains and growing conditions employed will affect both the amount and feed quality of Azolla biomass generated (Brouwer et al. 2018). To ascertain the potential for growing Azolla for protein feed, a simultaneous investigation of biomass productivity and chemical composition is required, as the meaningful output of a farming system is the result of both quantity and quality (Brouwer et al. 2018).

Animals can be given either fresh or dried Azolla. It can be given to cattle, poultry, sheep, goats, pigs, and rabbits either directly or in combination with their diet. The animals should be fed the concentrates early because it takes a few days for them to get used to the flavor of the Azolla. The plant should be carefully cleaned with fresh water to clear the dung odor when using dung as fertilizer in Azolla ponds (Hamidan et al. 2024).

## Azolla in ruminant diets

A growing problem for animal livestock production is finding affordable and sustainable protein sources (Bhujel and Rizal 2022). The production and processing of feed accounts for 45% of the cattle industry’s overall greenhouse gas emissions. The other sources of greenhouse gas emissions, which largely depend on feed types, include enteric methane (35%), land-use change (9%), and manure nitrous oxide and manure methane combined (9.5%) (Makkar 2016). Azolla fern is abundant and well-known to be a threat to the environment.

### Goat

*Azolla microphylla* is particularly suitable as a feed supplement for dairy cattle, due to its high nutritional content, especially crude protein and ether extract, both essential for enhancing milk production (Ting et al. 2022). Adding dried Azolla at 5% of the concentrate mixture in heifer diets has been reported to improve feed conversion efficiency (FCE) by 20% and average daily gain (ADG) by 15.7% (Roy et al. 2016). In buffalo, substituting sun-dried Azolla for 25% of the protein component of the concentrate mixture did not negatively impact nutrient digestibility (Kumari et al. 2021), while milk production and daily weight gain remained stable. Similarly, in Nellore sheep and buffalo, replacing 30% and 50% of groundnut protein with Azolla meal improved dry matter digestibility, daily growth rates, and feed efficiency Reddy et al. 2011). Furthermore, in goats, sun-dried Azolla can safely replace up to 15% of concentrate mixtures without adverse effects on feed economics (Acharya et al. 2023).

The nutritional potential of Azolla meal in a mixed total ration at different nutritional levels was evaluated on nutrient utilization and metabolic status of goats (Kumari et al. 2021), dried Azolla can add up to (15–25) % of the concentrate combination of growing goats to reduce the cost of diet. Osman badi goats can be fed Azolla meal up to 15% of the concentrate mixture instead of other foods because of its high fiber content (Korir 2024). *Azolla pinnata* contains high protein compounds and other components that may be useful in modulating body weight gain and plasma metabolites if fed to goats growing in subtropical regions (Al-Suwaiegh 2023). To determine the dietary effectiveness of Azolla feeding, a weaner lamb test was conducted, and the treatment groups showed increased dry matter and protein consumption with no negative impact on carcass features (Toradmal et al. 2017).

### Cattle, goats, and buffalo

Azolla supplementation in the feeding regimen of crossbred cows is beneficial, as evidenced by increased milk yield, milk fat yield, and a better benefit-cost ratio (Kour et al. 2020). The low-cost supplementation of Azolla can additionally increase the mean economic returns from a single cow by increasing milk yield each month in a village (Verma & Dey., 2021). *Azolla pinnata* can be employed as a novel approach to protein replacement in Sahiwal female calves (Bhatt et al. 2021). Additionally, adding 2 kg of azolla per day to the diets of crossbred cows increased milk and FCM yield by 11.2 and 12.5%, respectively, as well as feed conversion efficiency measured by kg DMI / kg feed conversion efficiency (FCM) yield (Arora et al. 2022). Additionally, several studies have shown that fresh Azolla can be added to animal feed up to 2 kg per day or substituted for dried Azolla up to 20% of commercial feed for dairy cows and buffalo, resulting in a 15%–20% reduction in commercial feed consumption and a 20% increase in milk production. Using Azolla in animal nutrition can enhance animal performance and reduce feeding expenses, we can conclude. (El Naggar and El-Mesery 2022).

*Azolla microphylla* is more suited to use as a supplement for dairy ruminants since it has a higher nutritional value in terms of crude protein and ether extract, both of which are required for the ruminant diet (Ting et al. 2022). According to Sihag et al. (2018), sun-dried Azolla can replace up to 15% of a concentrated mixture of goat feeds without having any negative effects on economic feeding. In buffalo, substituting sun-dried Azolla for 25% of the concentrate mixture’s protein did not significantly alter the animals’ ability to digest nutrients (Kumari et al. 2021). Additionally, Nellore sheep and buffalo showed better DM digestibility, daily growth, and feed efficiency when their diets used Azolla meal in place of 30 or 50% groundnut protein, respectively (Reddy et al. 2011). FCE increased by 20% and average daily gain (ADG) by 15.7% when dried Azolla was added at a 5% level to the concentrate mixture of the heifers’ diet (Roy et al. 2016).

### Sheep

Body weight, DM digestibility, and feed conversion ratio (FCR) were increased when Corriedale sheep’s diets were supplemented with 6% Azolla instead of 25% linseed cake (Korir 2024). FCR is a common parameter used in animal nutrition to measure how efficiently animals convert feed into desired output, usually body weight gain or milk production. Bhatt et al. (2021) found that, in the diet of Jalauni lambs, mustard cake protein was the best substitute for 25% of Azolla with no discernible impact on the nutrients’ digestion and utilization. According to a recent study by Singh et al. (2023), adding dry Azolla to lamb diets by up to 20% improved carcass characteristics. After six hours of feeding, animals fed sun-dried azolla began to exhibit considerably greater rumen characteristics (pH, VFA, and NH3-N) than those fed clover hay. *Azolla pinnata* is often sun-dried (Nayel et al. 2024).

## Azolla in non-ruminant diets

Azolla is regarded as the most economical and plentiful possible source of protein and opens up the possibility of lower production costs in the poultry industry (Alalade and Iyayi, 2008). From the perspective of the bird, Azolla contains tannins (Seid 2023). This can affect the digestion of proteins, so it’s critical to assess low dietary inclusion rates to figure out how much can be supplied safely (Seid 2023). Additionally, poultry diets that substitute low amounts of aquatic plants performed better, particularly when such plants provided a portion of the overall protein or acted as an egg pigment source (Gangadhar et al. 2015). There are numerous researchers worldwide who advocate for the usage of alternate high-quality protein source feed (Abate et al. 2020).

### Duck and fish

Farming ducks is one of the methods that farmers can employ to improve the sustainability and efficiency of land usage in rice fields. Duck production is one facet of an integrated farming system that is seen to be essential to sustainable agricultural growth (Palmario 2021). An integrated agricultural system is an attempt to use all available energy to produce it equitably. Using big squares to raise ducks, plant Azolla, and raise fish (Mustabi et al. 2021). Azolla-based diets enhanced the feed conversion ratio, performance index, average performance, egg production, egg shape index, egg weight, and yolk color of laying ducks (Alagawany et al. 2024). Feeding 55–75% Azolla under extensive production was a prospective possibility for Pekin duck raisers to reduce feed costs and, as a result, increase profit (Cabaral and Rieta 2020).

Integration of crops, livestock, and fish using Azolla plants as an alternative feed for ducks, fish, and fertilizer has increased farmer and livestock breeder income, reduced crop failure risk due to reliance on one commodity, can be in harmony with nature and is simple to implement because it is used locally (Mustabi et al. 2021). Feeding trials with species like tilapia (*Oreochromis niloticus*), catfish (*Clarias batrachus*), common carp (*Cyprinus carpio*), and freshwater prawn (*Macrobrachium rosenbergii*) showed improved growth performance, survival rates, and immunity. Azolla meal represents a promising solution to meet the growing demand for sustainable aquafeeds (Yohana et al. 2023).

### Broiler chicks

Traditionally, poultry production has been among the most lucrative, delivering high-quality meats and eggs for human consumption in the quickest amount of time (Parthasarathy 2024). Poultry, particularly ducks and chickens, can be fed a diet that includes fresh Azolla. The nutrient digestibility of crude protein, crude fat, and crude fiber was not changed by the level of Azolla in the ration, and broilers can readily digest the crude fiber in Azolla but not in rice bran, indicating that digestibility is not a limiting factor when Azolla is utilized (Joysowal et al. 2018).

*A. pinnata* supplementation enhances liver health, immune function, and growth performance in broilers through bioactive compounds. Its phenolic compounds (e.g., 5-Hydroxy-7-methoxyflavone) and flavonoids reduce oxidative stress by upregulating antioxidant genes like SOD1 and CAT, which protect liver tissue from damage (Hamouda et al. 2024). It increases the expression of immune-related genes (*IL8*, *IL10*, *TLR2*), enhancing disease resistance and inflammatory response regulation. They found that 10–15% Azolla protein meal (APM) supplementation for balanced benefits in liver protection, immune support, and growth, with no adverse effects on giblet weight or blood parameters.

Dried Azolla leaf meal controls muscle protein synthesis by activating the mammalian target of rapamycin/6S kinase signaling, and it can be added to a broiler chicken diet up to 5% without significantly affecting meat quality or performance (Abdelatty et al. 2020). Dietary intake of dried Azolla up to 12% can improve broiler chick growth performance, blood parameters, and antioxidant qualities while having no negative impact on the health of the chicks and increasing profitability (Kamel and Hamed 2021). Feeding Azolla to broilers increased carcass parameters, including gizzard weight, breast muscle yield, and lower meat pH (Shambhvi et al. 2021).

In conclusion, Azolla offers a low-cost, eco-friendly solution to global challenges in food security, pollution, and energy sustainability, aligning with greener agricultural and industrial practices. Azolla is a nutritious feed supplement for livestock, particularly poultry and fish, due to its high protein content, vitamins, and minerals. It contains about 26% crude protein, 16% crude fiber, and essential minerals, making it suitable for cattle, fish, and poultry. Azolla feed enhances milk productivity in cattle by approximately 15% when about 2 kg of Azolla is fed per day along with regular feed. For poultry, it improves the weight of broiler chickens and increases egg production in layers. *A. pinnata* is a natural, multifunctional feed additive that supports broiler health and productivity through antioxidant and immunomodulatory mechanisms. In the future, we will use it to evaluate its efficiency as a food source for rabbits.

## Data Availability

The data supporting the findings of this study are available from the corresponding author upon reasonable request.
